# Regional Features of MuSK Antibody-Positive Myasthenia Gravis in Northeast China

**DOI:** 10.3389/fneur.2020.516211

**Published:** 2020-10-02

**Authors:** Zunwei Zhang, Yujia Guan, Jiale Han, Mingming Li, Miao Shi, Hui Deng

**Affiliations:** ^1^Department of Neurology and Neuroscience Center, First Hospital of Jilin University, Changchun, China; ^2^Department of Endocrinology, First Hospital of Jilin University, Changchun, China

**Keywords:** muscle specific receptors tyrosine kinase, acetylcholine receptor, myasthenia gravis, ocular muscle, tacrolimus

## Abstract

**Objective:** To summarize the characteristics of muscle-specific receptor tyrosine kinase antibody-positive myasthenia gravis (MuSK-MG) in Northeast China.

**Methods:** We retrospectively collected 183 confirmed MG patients and divided them into three groups based on the type of serum antibodies: MuSK-MG (14 cases), acetylcholine receptor (AChR)-MG (130 cases), and double-seronegative (DSN)-MG (39 cases). The clinical, diagnostic, therapeutic, and prognosis data were analyzed.

**Results:** MuSK antibody was detected in 26.7% of seronegative MG. The mean age of onset in MuSK-MG was 53.2 ± 13.6 years. Fifty percent of MuSK-MG patients with an onset symptom of pure ocular muscle weakness. The time from onset to other muscle groups' involvement and the time from onset to myasthenic crisis had no significant difference among the three groups (*P* > 0.05). The proportion of Osserman classification I in MuSK-MG group was lower than that in DSN-MG group. The proportion of Osserman classification IV in MuSK-MG group was higher than that in the other two groups. The incidences of other coexisting autoimmune diseases in MuSK-MG group were higher. Prognosis after the treatment of steroid combined with tacrolimus for MuSK-MG was similar to AChR-MG treated with steroid combined with an immunosuppressant agent (*P* > 0.05).

**Conclusion:** Patients with MuSK-MG in Northeast China have a modestly later onset age and a proportion of patients may have a mild form of the disease with delayed disease progression. We confirmed the existence of a rare ocular MuSK-MG phenotype, a high proportion of coexisting with other autoimmune diseases, and a good response to steroids combined with tacrolimus for our MuSK-MG series.

## Introduction

Myasthenia gravis (MG) is an autoimmune disorder caused by antibodies targeting proteins associated with neuromuscular junction (NMJ) transmission (1). The most common antibodies are against acetylcholine receptor (AChR). Such patients are called AChR-MG. However, 10–20% of MG patients, often termed seronegative MG (SNMG), do not have serum AChR antibodies (Ab). A proportion of those patients have relatively mild manifestations, whereas another subgroup of patients have generally severe disease with severe respiratory and bulbar muscle weakness. In 2001, Hoch et al. confirmed the presence of muscle-specific receptor tyrosine kinase (MuSK) Ab in 70% of SNMG patients (2). MuSK Ab exerts a dose-dependent block of MuSK binding to ColQ, leading to the reduction of ColQ binding to NMJ. The lack of ColQ in NMJ can compromise agrin-mediated AChR clustering (3).

MuSK Ab-positive MG (MuSK-MG) has been defined as a distinct MG sub-group, which often leads to more severe muscle weakness (2). Some patients lack AChR Ab and MuSK Ab (double-seronegative MG, DSN-MG), and often have a mild manifestation, which seems to explain the phenomenon of polarized clinical features in SNMG. However, not all MuSK-MG patients have severe clinical features. Previous studies have found that the clinical presentation of MuSK-MG may vary by region and race. For example, the positivity for MuSK Ab in SNMG patients were 40% in Mediterranean countries (4), whereas in Asian countries it is 20–30% (5). In Europe and the USA, most MuSK-MG patients onset before 40 years (6). However, the onset age of MuSK-MG patients was slightly older ranging from 40 to 50 years in Japan (7), South Korea (8), and Taiwan (9). Initially, rare reports from western countries suggested that pure ocular muscle weakness at onset of MuSK-MG can occur (10, 11). On the contrary, it was the only onset symptom in Taiwan (9). Moreover, recent reports on ocular MuSK-MG are also increasing worldwide (12–18). In addition, previous studies have not reached consensus on immunotherapy option of MuSK-MG. Some scholars recommend the first choice of rituximab (19). Therefore, the specific clinical information and treatment of MuSK-MG patients still need further observation and investigation.

This study retrospectively analyzed the clinical data of MuSK-MG patients in Northeast China, and reviewed the previous literature to summarize the clinical feature and diagnosis as well as the therapeutic management of MuSK-MG in this area, so as to improve the clinicians' further understanding of the disease.

## Methods

### Patient Information

We retrospectively collected 183 confirmed MG patients who were admitted to the First Hospital of Jilin University from January 1, 2015 to December 31, 2019. According to the type of serum antibodies, the patients were classified into three groups, MuSK-MG (14 cases), AChR-MG (130 cases), and DSN-MG (39 cases). The clinical, diagnostic, therapeutic, and prognosis data of these patients, including gender, age of onset, initial symptom, disease progression, clinical classification, disease severity, pharmacological, electrophysiological, and serological findings, results of thymus examination, comorbidities, therapeutic options, and prognosis, were analyzed.

Incomplete information was completed at outpatient clinic visits. Each patient gave informed consent. The study was approved by the ethics committee of the First Hospital of Jilin University.

### Diagnosis Criteria

The diagnosis of MG was confirmed based on typical clinical features of fluctuating muscle weakness, and at least one of the following positive tests: a positive pharmacological response on intramuscular injection of neostigmine; decrement of >10% on repetitive nerve stimulation (RNS) test; AChR Ab or MuSK Ab positive.

### Disease Severity Assessment

The clinical status and disease severity were evaluated according to the Osserman classification and quantitative MG score (QMGs), respectively (20).

### Neostigmine Trial

First, the initial disease severity of a patient was evaluated by full QMGs. After a 10-min rest, appropriate dose (0.02–0.03 mg/kg body weight) of neostigmine was administered. To relieve the possible muscarinic side effect, atropine (0.5 mg) was administered before the neostigmine injection. Then, disease severity was evaluated by QMGs at 10-min intervals until 60 min after the neostigmine injection. The most significant improvement in QMGs was calculated: (QMGs before the injection – minimal QMGs)/QMGs before the injection ×100%. The results of the neostigmine trial were categorized into positive (>60%), probable positive (25–60%), or negative (<25%).

### Repetitive Nerve Stimulation

The methods measured by RNS are as follows: First, routine nerve conduction studies were performed to exclude peripheral neuropathy. Next, repetitive stimulation was performed in selected muscles at a rate of 2–5 times and 10–50 times per second, respectively. A compound muscle action potential (CMAP) of the fourth to fifth wave decrement of >10% compared with the first wave was considered abnormal. In addition, a CMAP increment of >100% was also regarded as abnormal.

### Antibody Testing

MG-related serum antibodies were tested using a commercial ELISA kit (Euroimmun, Lübeck, Germany). All patients were tested for AChR Ab titers before receiving immunosuppressive therapy. If the AChR Ab were negative, MuSK Ab is further tested. One hundred microliters of patient serum was diluted at 1:100 into the microtiter well-plate. The microtiter plate was incubated for 60 min at room temperature (RT) on an orbital shaker (500 rpm) and then washed three times with 250 μl of diluted wash buffer. One hundred microliters of antiserum was pipetted in each well and incubated in a microtiter plate for 60 min at RT on an orbital shaker (500 rpm). The incubation solution was discarded and the plate was washed three times with 250 μl of diluted wash buffer. One hundred microliters of *p*-nitrophenyl phosphate (PNPP) substrate solution was pipetted into each well and incubated in a microtiter plate for 30 min at RT. The substrate reaction was stopped by adding 100 μl of PNPP stop solution into each well, and then the optical density was measured with a photometer at 405 nm.

### Therapy

Therapeutic strategies mainly consist of symptomatic treatment and immunosuppressive therapy. Symptomatic treatment with oral pyridostigmine bromide was performed to the patients who responded positive on the neostigmine trial. Corticosteroids were combined with an immunosuppressive agent (azathioprine, tacrolimus, mycophenolate mofetil, cyclophosphamide). All of the follow-up MuSK-MG patients were prescribed with tacrolimus as an immunosuppressant. During the treatment period, the dosage of steroid was gradually increased or decreased according to the patient's condition, and the patient was given corresponding supportive treatment to prevent the corticosteroid side effects. Intravenous immunoglobulin (IVIg) and plasma exchange was given according to the patient's condition and economic affordability.

### Prognosis

The QMG score of a patient at the first visit was regarded as baseline. Follow-up clinic visits were performed at the first and third months after initial visit and 3-month intervals thereafter, and a QMG score was evaluated at each visit and compared with the baseline QMG score: (baseline QMG score – a follow-up QMG score)/baseline QMG score ×100%. A degree of improvement >95% was classified as cured, 80~95% was basically cured, 50–79% was markedly effective, 25–49% was effective, and <25% was ineffective, respectively. This method was originated from the clinical absolute and relative score system for MG proposed by Professor Xu Xianhao in 1993 (21) and has been largely used in China.

### Statistical Analysis

SPSS 22.0 statistical software was used for data statistics. Normally distributed data analysis was performed by Student *t-*test. Non-normally distributed data analysis was performed by Mann–Whitney *U*-test. For categorical data, χ^2^ test or Fisher exact test was used for analyzing the difference between groups. *P* < 0.05 was considered statistically significant.

## Results

Detailed clinical data of the 14 MuSK-MG patients are presented in Tables 1A,B. The mean time of patient follow-up was 11.8 ± 11.0 months, ranging 1–42 months.

**Table 1A:** Detailed clinical data of the 14 MuSK-MG patients.

**Patient number**	**1**	**2**	**3**	**4**	**5**	**6**	**7**
Sex	Female	Female	Female	Male	Female	Female	Female
Onset age (years)	53	62	71	70	63	34	64
MuSK Ab titer (U/ml)	4.45	>12.00	>12.00	3.63	5.48	5.47	1.01
Disease duration (months)	17	74	4	96	78	25	38.5
Onset symptom	Ptosis, slurred speech	Dysphagia, slurred speech	Dyspnea	Slurred speech, dysphagia	Slurred speech	Diplopia	Limb weakness
Tongue muscle atrophy	Yes	Yes	No	Yes	No	No	No
Jolly test	Positive	Negative	Positive	Positive	Positive	Positive	Positive
Time from onset to involvement of other muscle groups (months)	10.5	71	2	90	60	–	12
Most severe Osserman classification	IIb	IIb	III	IIb	IV	I	IV
Maximum QMG scores	13	21	22	10	24	4	24
Time from onset to the peak (months)	17	72	3.2	96	73.5	0.3	37.5
Number of myasthenic crisis	0	0	1	0	1	0	2
Neostigmine trial	Positive	Negative	Negative	Positive	Positive	Positive	Negative
Cholinergic side effect	Yes	Yes	Yes	No	Yes	No	Yes
Decrement on RNS (3 Hz)	Yes	Yes	Yes	Not done	Not done	No	No
Thymus CT scan/pathology	Normal	Normal	Normal	Normal	Normal	Normal	Normal
TG/TPO Ab	Normal	Increased	Increased	Normal	Increased	Normal	Increased
Other AD	Hyperthyroidism	No	Hashimoto thyroiditis	No	No	Behcet's disease	Hashimoto thyroiditis
Intravenous immunoglobulin	Yes	No	No	No	Yes	No	Yes
Glucocorticoid	Yes	Yes	Yes	Yes	Yes	Yes	Yes
Tacrolimus	Yes	Yes	Yes	Yes	Yes	Yes	No
Pyridostigmine bromide	30 mg tid	No	No	60 mg tid	No	60 mg tid	No
Follow-up time (months)	12	12	21	3	1	42	0.25
QMG scores after treatment	5	4	5	4	15	0	–
Prognosis	Markedly effective	Basically cured	Markedly effective	Markedly effective	Effective	Cured	Died

**Table 1B:** Detailed clinical data of the 14 MuSK-MG patients.

**Patient number**	**8**	**9**	**10**	**11**	**12**	**13**	**14**
Sex	Female	Male	Female	Female	Female	Male	Female
Onset age (years)	64	51	62	55	50	32	29
MuSK Ab titer (U/ml)	1.08	1.20	>12.00	5.90	5.18	1.40	>12.00
Disease duration (months)	4	2	54	135.6	3	70	1
Onset symptom	Ptosis, diplopia	Ptosis	Diplopia	Ptosis, diplopia	Diplopia	Ptosis, diplopia	Ptosis, dysphagia
Tongue muscle atrophy	No	No	No	No	No	No	No
Jolly test	Positive	Positive	Positive	Negative	Negative	Positive	Positive
Time from onset to involvement of other muscle groups (months)	3.5	1.5	3	132	7	6	1
Most severe Osserman classification	IIb	IIb	IV	IIb	IIa	IIb	IIb
Maximum QMG scores	14	11	22	12	4	20	13
Time from onset to the peak (months)	4	2	54	135.6	7	7	1
Number of myasthenic crisis	0	0	2	0	0	0	0
Neostigmine trial	Positive	Positive	Negative	Negative	Positive	Positive	Positive
Cholinergic side effect	No	No	Yes	Yes	No	No	No
Decrement on RNS (3 Hz)	Not done	No	Yes	Yes	Yes	Yes	Yes
Thymus CT scan/pathology	Normal	Normal	Normal	Normal	Bronchogenic cyst	Normal	Normal
TG/TPO Ab	Yes	Yes	Yes	Yes	No done	No done	No done
Other AD	Psoriasis	Allergic dermatitis	Without	Without	Without	Without	Without
Intravenous immunoglobulin	No	No	Yes	Yes	No	No	Yes
Glucocorticoid	Yes	Yes	Yes	Yes	Yes	Yes	Yes
Tacrolimus	Yes	Yes	Yes	Yes	Yes	Yes	Yes
Pyridostigmine bromide	60 mg tid	60 mg tid	No	No	60 mg tid	60 mg tid	60 mg tid
Follow-up time (months)	3	3	3	12	12	12	18
QMG scores after treatment	7	3	10	2	2	4	4
Prognosis	Markedly effective	Markedly effective	Markedly effective	Basically cured	Markedly effective	Basically cured	Markedly effective

*MuSK-Ab (U/ml) (cut-off >0.40); QMG, quantitative myasthenia gravis; tid, three times a day; TG Ab, thyroglobulin antibody; TPO Ab, thyroid peroxidase antibody; AD, autoimmune disease; RNS, repetitive nerve stimulation*.

### Demographic Data of MuSK-MG Patients

Anti-MuSK antibodies were detected in 26.4% of SNMG. Females predominated in both MuSK-MG (11/14, 78.6%) and AChR-MG (82/130, 63.1%). On the contrary, DSN-MG showed a marked male predominance (24/39, 61.5%). The mean onset age of MuSK-MG was 53.2 ± 13.6, which is not different from AChR-MG group and DSN-MG group (*P* = 0.876, *P* = 0.080) (see Table 2).

**Table 2:** Basic information of the three groups.

**Groups**	**MuSK-MG (*n* = 14)**	**AChR-MG (*n* = 130)**	**DSN-MG (*n* = 39)**
Age at onset (years)	53.2 ± 13.6	53.9 ± 16.7	45.0 ± 15.2
Gender (male/female)	3:11	48:82	24:15

Tables 3A–C summarizes the clinical features, diagnostic tests, and comorbidities or coexisting antibodies for the three groups of patients.

**Table 3:** Clinical, diagnostic tests, complications, and laboratory information of three groups of patients.

**Groups**		**MuSK-MG**	**AChR-MG**	**DSN-MG**	***P* value**
**(A) Clinical features**					
Onset distribution	Pure ocular	7/14 (50.0%)	98/130 (75.4%)	30/39 (76.9%)	0.107
	Pure limb	1/14 (7.1%)	10/130 (7.7%)	5/39 (12.8%)	>0.05
	Pure bulbar	3/14 (21.4%)	6/130(4.6%)	1/39(2.6%)	<0.05^*^
	Respiratory	1/14 (7.1%)	3/130 (2.3%)	-	0.339
	Oculobulbar	2/14 (14.3%)	13/130 (10.0%)	3/39 (7.7%)	>0.05
Positive rate of Jolly test		11/14 (78.6%)	130/130 (100%)	39/39 (100%)	<0.001^***^
Tongue muscle atrophy		3/14 (21.4%)	1/130 (0.8%)	-	0.003^**^
Most severe Osserman classification	I	1/14 (7.1%)	38/130 (29.2%)	23/39 (59.0%)	<0.001^***^
	IIa	1/14 (7.1%)	20/130 (15.4%)	4/39 (10.3%)	0.545
	IIb	8/14 (57.1%)	61/130 (46.9%)	11/39 (28.2%)	0.068
	III	1/14 (7.1%)	8/130 (6.2%)	1/39 (2.6%)	0.660
	IV	3/14 (21.4%)	3/130 (2.3%)	-	<0.05^*^
**(B) Diagnostic tests**					
Neostigmine trial		9/14 (64.3%)	108/109 (98.2%)	33/34 (97.1%)	<0.05^*^
Cholinergic side effect		7/14 (50.0%)	2/109 (1.8%)	-	<0.001^***^
Decrement on RNS (3 Hz)		8/11 (72.7%)	65/88 (73.9%)	16/28 (57.1%)	0.238
**(C) Coexisting other AD/Abs**					
Thymic abnormalities		1/14 (7.1%)	58/130 (44.6%)	10/39 (25.6%)	0.006^**^
Other AD		6/14 (42.9%)	19/130 (14.6%)	7/39 (17.9%)	0.030^*^
TG/TPO Ab		8/11 (72.7%)	35/93 (37.6%)	10/23 (43.5%)	>0.05

Comparison of clinical, diagnostic tests, complications and laboratory examination data among the three groups was done by χ^2^ test or Fisher exact χ^2^ test.

* indicates P < 0.05,

** indicates P < 0.01,

*** indicates P < 0.001.

*The data are shown as mean ± SD or ratio. AD, autoimmune disease; RNS, repetitive nerve stimulation; TG, thyroglobulin; TPO, thyroid peroxidase; Ab, antibody*.

### MuSK-MG With a High Rate of Ocular Muscle Weakness at Onset

Weakness of muscle involvement at onset for MuSK-MG patients was classified into ocular (7/14, 50.0%), bulbar (3/14, 21.4%), respiratory (1/14, 7.1%), limb (1/14, 7.1%), and oculobulbar muscles (2/14, 14.3%). Patients with MuSK-MG had a high rate pure bulbar muscle weakness at onset compared with those with AChR-MG (*P* = 0.044). There were no significant differences in other muscle involvement at onset among the three groups (*P* > 0.05) (Table 3A).

### Atypical Clinical Feature and Tongue Muscle Atrophy in MuSK-MG

Compared with AChR-MG and DSN-MG groups, the positive rate of Jolly test in MuSK-MG group was lower (*P* < 0.05). Tongue muscle atrophy in patients with MuSK-MG was more frequent than AChR-MG and DSN-MG patients (*P* < 0.05) (Table 3A).

### A Similar Disease Progression Between MuSK-MG and AChR-MG

**Figure 1:**
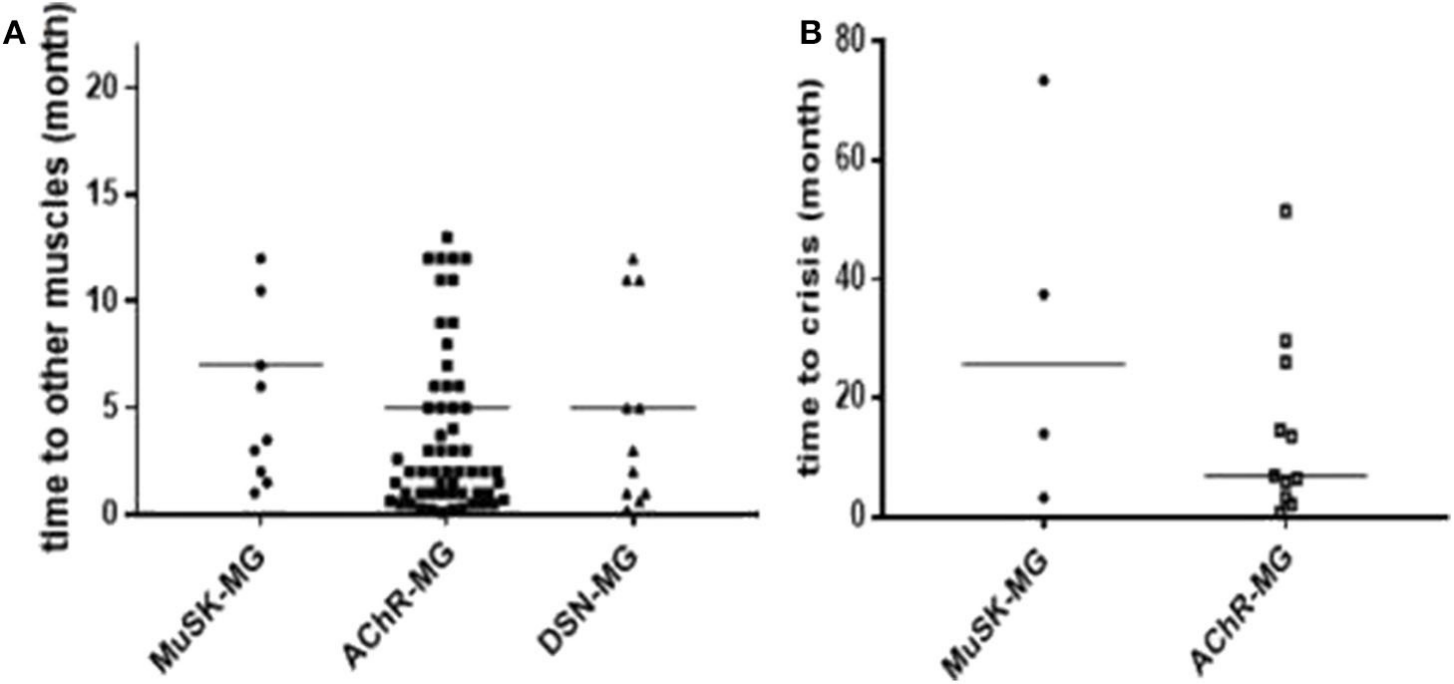
Comparison of disease progression in each group. **(A)** There was no significant difference in the time from onset to other muscle group involvement among the three groups. **(B)** There was no significant difference in the time from onset to myasthenic crisis between MuSK-MG group and AChR-MG group. Comparison between groups was done by Mann-Whitney test.

The median time from onset to other muscles' involvement among the three groups had no significant difference (Figure 1A). In addition, the median time from onset to myasthenic crisis was 25.75 (5.9, 64.5) months in MuSK-MG group, which was not different from AChR-MG group (*P* = 0.267) (Figure 1B).

### Ocular and Late Severe Phenotype MuSK-MG

As shown in Table 3A, the proportion of Osserman classification I between MuSK-MG group and AChR-MG group had no significant difference (*P* = 0.147); however, such proportions in both MuSK-MG and AChR-MG groups were much lower than that in DSN-MG group (*P* = 0.001, *P* = 0.001). Compared with the AChR-MG group and DSN-MG group, the proportion of Osserman classification IV in MuSK-MG group was higher (*P* = 0.007, *P* = 0.016).

**Figure 2:**
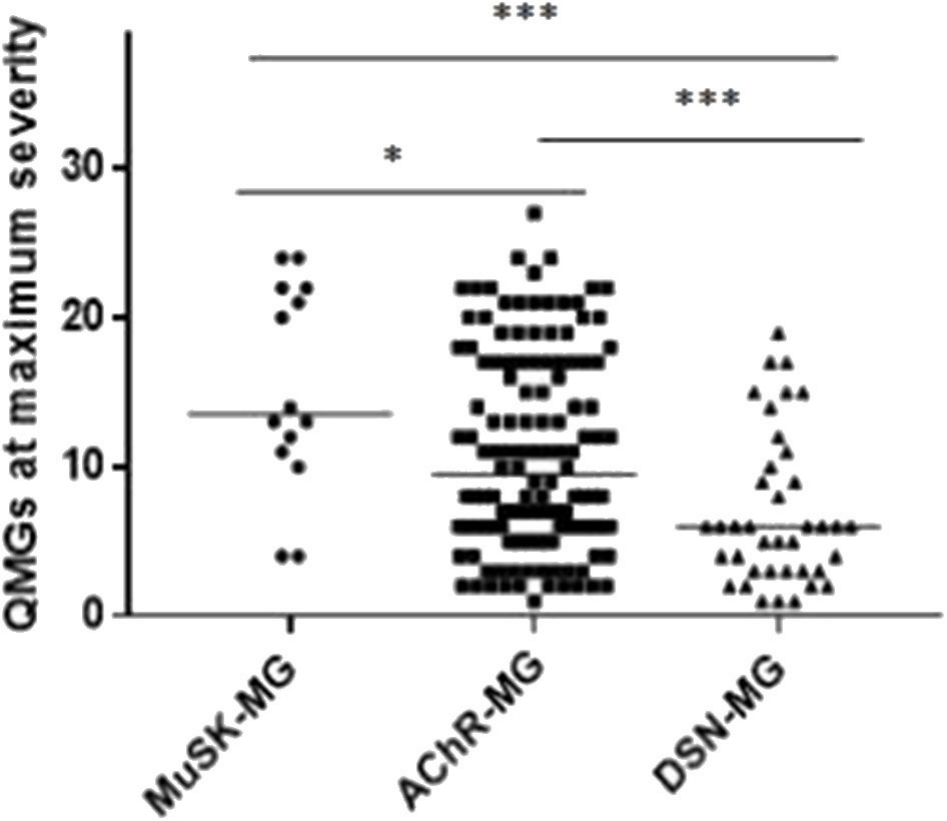
Comparison of maximum QMGs in each group. The maximum QMGs in MuSK-MG group was more severe than that in AChR-MG group and DSN-MG group. Moreover, QMGs in AChR-MG group was significantly higher than that in DSN-MG group. Comparison between groups was done by Mann-Whitney test. * indicates *P* < 0.05, *** indicates *P* < 0.001.

Figure 2 shows the maximum QMGs of the patients in each group. The QMGs in the MuSK-MG group were more severe than that in AChR-MG group and DSN-MG group (*P* = 0.023, *P* < 0.001). In addition, the QMGs in the AChR-MG group was also significantly higher than that in DSN-MG group (*P* = 0.001).

### Diagnostic Testing

The positive rate of neostigmine trial in MuSK-MG group was lower than that in AChR-MG group and DSN-MG group (*P* < 0.001, *P* = 0.008). The incidence of cholinergic side effects in MuSK-MG group was significantly higher than that in AChR-MG group and DSN-MG group (*P* < 0.001, *P* < 0.001) (Table 3B).

The positive rate of RNS decrement (3 Hz) among the three groups was not significantly different (*P* = 0.238) (Table 3B).

As shown in Table 1, the auxiliary examinations for the diagnosis of MuSK-MG included pharmacological tests, neurophysiological examinations, and serological tests. Among them, four patients were positive for all three auxiliary examinations, nine patients were positive for two of the three auxiliary examinations, and one patient had merely MuSK antibody positive as the basis for diagnosis.

### Complications

**Figure 3:**
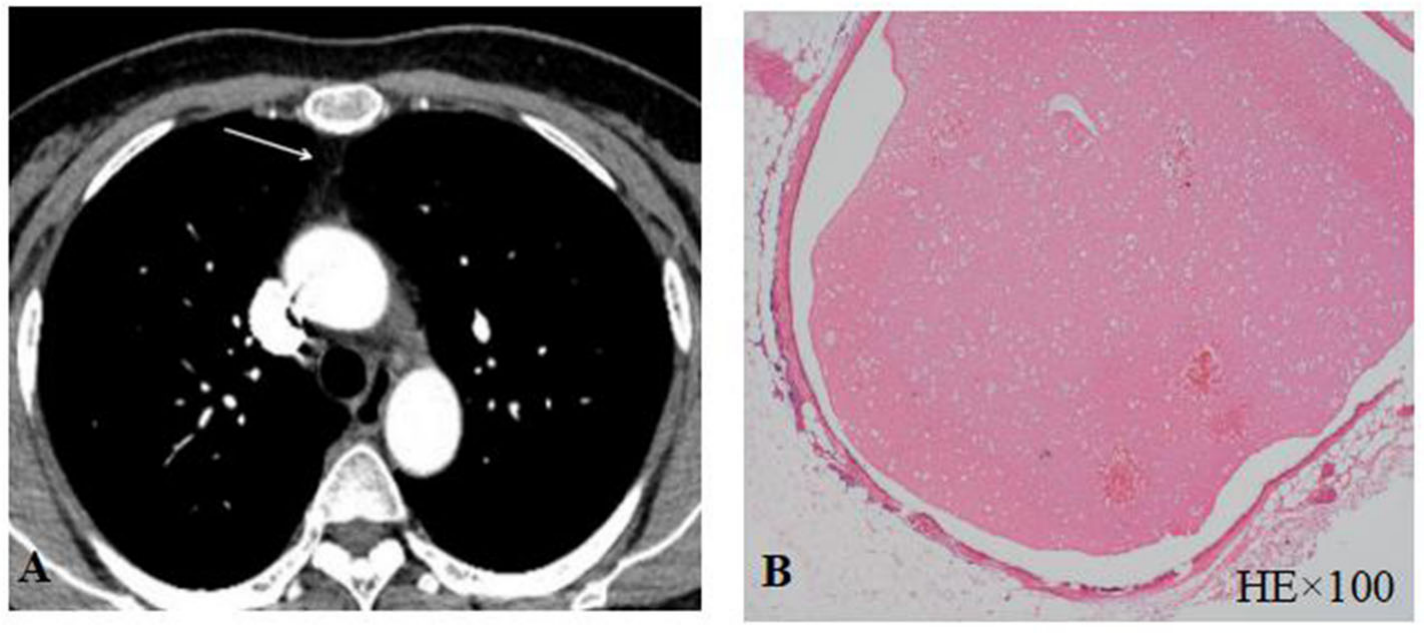
**(A)** Thymus CT scan revealed a small nodule in the thymus area; **(B)** thymic bronchogenic cyst was shown by HE staining.

Thymic CT scan and/or thymic pathological examination was performed in all enrolled patients. Only one patient (#12) in MuSK-MG group had a small nodule in the thymus region (Figure 3A). The pathological report of thymus indicated that the lesion was thymic bronchogenic cyst (Figure 3B). In contrast, 42 patients had thymoma and 16 patients had thymic hyperplasia in AChR-MG group. Five out of 39 (12.8%) patients with DSN-MG had thymoma; another 5 out of 39 (12.8%) patients with DSN-MG had thymic hyperplasia. The proportion of thymus abnormalities in the MuSK-MG group was lower than that in AChR-MG group (*P* = 0.007), whereas it did not differ in the DSN-MG group (*P* = 0.280) (Table 3C).

The frequency other autoimmune disease (AD) in MuSK-MG was higher than that in AChR-MG group (*P* = 0.023), but not different from DSN-MG group (*P* = 0.135). In MuSK-MG group, patient 1 coexisted with hyperthyroidism, patient 3 and patient 7 coexisted with Hashimoto thyroiditis, patient 6 coexisted with Behcet's disease, patient 8 coexisted with psoriasis, and patient 9 coexisted with allergic dermatitis.

The positive rates of thyroglobulin (TG) and/or thyroid peroxidase (TPO) antibodies among the three groups were not statistically different (*P* > 0.05), although it was higher in the MuSK-MG group (Table 3C).

### Treatment and Prognosis

As shown in Table 4, 8 out of 14 MuSK-MG patients showed a good response to pyridostigmine bromide therapy, and this proportion was lower than that in AChR-MG group and DSN-MG group (*P* < 0.001, *P* < 0.001). The rates of treatment with glucocorticoid, immunosuppressants, and IVIg in MuSK-MG group were not different from those in the AChR-MG group (*P* = 0.051, *P* = 0.099, and *P* = 0.356), but higher than those in DSN-MG group (*P* < 0.001, *P* < 0.001, and *P* = 0.046). Plasma exchange was performed only in two AChR-MG patients. No patients received rituximab in our study.

**Table 4:** Therapeutic strategy among the three groups.

**Groups**	**MuSK-MG**	**AChR-MG**	**DSN-MG**
Pyridostigmine bromide	8/14 (57.1%)	130/130 (100%)	39/39 (100%)
Glucocorticoid	14/14 (100%)	94/130 (72.3%)	16/39 (33.3%)
Immunosuppressant	13/14 (92.9%)	86/130 (66.2%)	1/39 (2.6%)
Intravenous immunoglobulin	6/14 (42.9%)	37/130 (28.5%)	5/39 (12.8%)
Plasma exchange	–	2/130 (1.6%)	–

Two patients died from MG during the follow-up, including one MuSK-MG patient (patient 7 died in the first week after the initial visit) and one AChR-MG patient, and the mortality between the two groups had no statistical difference (*P* = 0.186).

Thirteen MuSK-MG patients, with an average follow-up time of 11.8 ± 11.0 (range 1–42) months, received treatment with prednisone (1 mg/kg body weight daily, tapered to 5 mg every 1–2 weeks) combined with tacrolimus (3 mg/day).

Treatment with prednisone plus an immunosuppressant (tacrolimus 3 mg/day, cyclophosphamide 100 mg/day, mycophenolate mofetil 1.0–2.0 g/day, or azathioprine 2–3 mg/kg body weight daily) was performed in 88 out of 130 AChR-MG patients.

**Figure 4:**
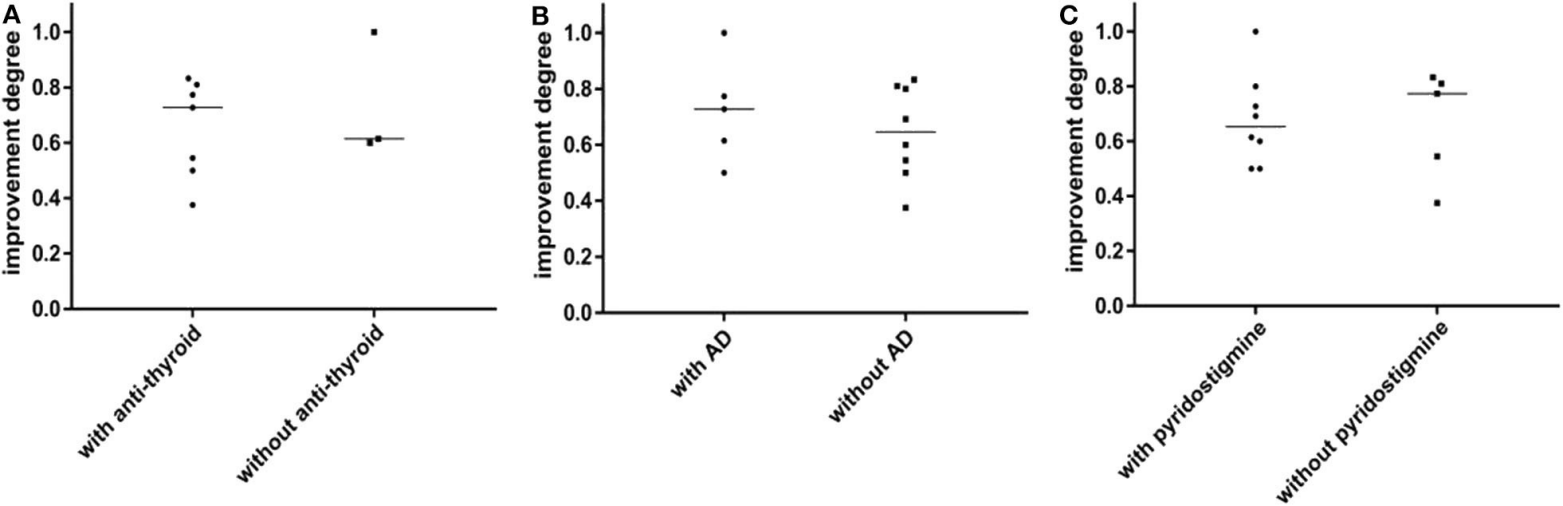
**(A)** The prognosis between MuSK-MG with thyroid antibodies and without thyroid antibodies has no significant difference. **(B)** The prognosis between MuSK-MG patients coexist with other autoimmune disease and without other autoimmune disease has no statistical difference. **(C)** There was no statistical difference in the prognosis between MuSK-MG patients who were treated with pyridostigmine bromide and those who were not. Comparison between groups was done by Mann-Whitney test. *P* < 0.05 indicated a statistically significant difference.

The prognosis between MuSK-MG group and AChR-MG group (mean follow-up time was 26.9 ± 14.8 (range 3–58) months) was not significantly different (Table 5). Most patients in the DSN-MG group only received symptomatic treatment with pyridostigmine bromide; thus, the analysis of prognosis comparison was not included. In addition, the prognosis of MuSK-MG patients in several special situations were also compared in this study (see Figure 4). The prognosis (degree of QMGs improvement) in MuSK-MG patients with thyroid antibodies [73.0% (50.0%, 81.0%)] compared with that of MuSK-MG patients without thyroid antibodies (62.0% (61.0%, 81.0%)] had no significant difference (*P* = 0.569) (Figure 4A).

**Table 5:** Comparison of the prognosis after steroid combined with immunosuppressive therapy between MuSK-MG and AChR-MG group.

**Groups**	**MuSK-MG**	**AChR-MG**	***P-*value**
Cured	1/13 (7.7%)	5/88 (5.7%)	0.572
Basically cured	3/13 (23.1%)	24/88 (27.3%)	1.000
Markedly effective	7/13 (53.8%)	45/88 (51.1%)	1.000
Effective	2/13 (15.4%)	14/88 (15.9%)	1.000
Ineffective	0	0	–

*Comparison of prognosis between MuSK-MG and AChR-MG was done by Fisher exact χ^2^ test. Significance was set at P < 0.05. The data are shown as ratio*.

In addition, the prognosis of MuSK-MG patients in several special situations was also compared in this study (see Figure 4). The prognosis (degree of QMGs improvement) in MuSK-MG patients with thyroid antibodies [73.0% (50.0%, 81.0%)] compared with that of MuSK-MG patients without thyroid antibodies [62.0% (61.0%, 81.0%)] had no significant difference (*P* = 0.569) (Figure 4A). Moreover, the prognosis of MuSK-MG patients with co-existence of other AD [73.0% (56.0%, 88.5%)] compared with that of MuSK-MG patients without AD [64.5% (51.25%, 80.75%)] had no significant difference as well (*P* = 0.628) (Figure 4B). The prognosis between MuSK-MG patients treated with pyridostigmine bromide [62.0% (50.0%, 73.0%)] and those of patients without pyridostigmine bromide [73.5% (50.25%, 80.75%)] was also not different (*P* = 0.567) (Figure 4C).

## Discussion

### Demographic Characteristics

We showed that the MuSK-MG patients represented 26.4% of SNMG patients. The prevalence of MuSK-Ab was similar to the frequency as found in Japan (7) and South Korea (8). Some studies reported that patients with MuSK-MG showed a more female predominance compared with the other two groups (22, 23). Another study reported that women predominated in all groups (23). However, in our results, MuSK-MG and AChR-MG patients showed a similar female predominance, but DSN-MG patients were predominantly male. The onset age of MuSK-MG was not statistically different from the other two groups, which was consistent with previous studies (23, 24). Interestingly, however, the mean age at onset in our MuSK-MG series was 53.2 ± 13.6 years, which was different from most Western countries. The onset age of MuSK-MG patients is prominent in the fourth decade (6). However, the onset ages of MuSK-MG in Japan (7), South Korea (8), and China's Taiwan (9) are mostly between 45 and 50 years. Compared with the earlier results, the onset age of MuSK-MG patients was even later in Northeast China.

### Clinical Features

In this study, ocular muscle weakness was the most common onset symptom in the three groups. Although the proportion of pure bulbar muscle weakness at onset in MuSK-MG (21.4%) was higher than that of AChR-MG, such a proportion is far lower than the proportion of 60.1% found by Baggi et al. (24). In addition, interestingly, our results showed no differences in the time from onset to the involvement of other muscle groups and the time from onset to myasthenic crisis among three groups, which was different from the previous reports of the rapid progress for MuSK-MG (10). Four MuSK-MG patients manifested mild symptoms for a long time. Patient 4 only showed mild dysarthria and remained stable for about 8 years. He then came to the hospital because of diplopia. The main manifestation of patient 5 was mild fluctuating slurred speech, and the symptoms lasted for more than 6 years. Then she came to the hospital due to limb weakness. Patient 11 initially showed ptosis and diplopia only, which lasted for 11 years, and she came to our hospital because of slurred speech. Patient 6 had purely ocular symptoms for more than 2 years; the Osserman classification of patient 6 was graded I. Therefore, the clinical progression in some of our MuSK-MG patients seems to be mild. In addition, ocular MG may be the fourth clinical phenotype for MuSK-MG, which differs from the typical three generalized phenotypes (18). Despite the subset of MuSK-MG patients with slow disease progression and relatively mild symptoms, the overall disease severity was higher in patients with MuSK-MG than that of the other two groups, consistent with previous studies (24).

### Diagnostic Testing

In our cohort, MuSK-MG had a lower positivity rate to neostigmine trial and a higher prevalence of cholinergic side effects compared with the other two groups, which were in accord with previous studies (25). Moreover, Wolfe et al. found that the overall positive rates of RNS between MuSK-MG and AChR-MG were not significantly different, but higher than that of DSN-MG (25). In this study, DSN-MG did have a relatively low positive rate of RNS, but the difference was not statistically significant compared with the other two groups. In addition, interestingly, one of 14 patients (patient 7) in this study lacked positive evidence of both neostigmine test and low-frequency RNS test, but this patient had the typical fluctuation of muscle weakness. The antibody testing result of this patient indicated that the MuSK-Ab titer was 1.01 U/ml (cut-off >0.40). After the immunosuppressive experimental treatment, the condition of the patient improved, and the patient was diagnosed as definite MuSK-MG. However, a more sensitive test—single-fiber electromyography—was not performed in this study, which limits the diagnostic efficacy of neurophysiological tests.

### Atypical Clinical Feature and Tongue Muscle Atrophy

Fluctuating skeletal muscle weakness is considered to be the typical clinical feature of MG. Interestingly, in this study, 21.4% (3/14) of MuSK-MG patients lacked the fluctuation of muscle weakness, which was different from the other two groups with a positive rate of 100% to Jolly test. Basic research has found that the presynaptic acetylcholine (ACh) release was increased in patients with AChR-MG, which is a compensation mechanism for failure of neuromuscular transmission caused by AChR loss (26). Therefore, most of the AChR-MG patients have fluctuating muscle weakness. However, such a compensation mechanism was not detected in MuSK-MG (27). The levels of presynaptic ACh release were low in MuSK-MG, and the miniature endplate potentials were small at the same time, which may explain why some MuSK-MG patients have no obvious fluctuation of muscle weakness.

However, not all MuSK-MG patients lack the typical fluctuating characteristics; hence, more research is still needed to explore for this issue.

In addition, we found that three MuSK-MG patients (1, 2, and 4) have tongue muscle atrophy, which showed a higher frequency of tongue muscle atrophy in MuSK-MG compared with AChR-MG and DSN-MG. In particular, the disease courses in those patients were insidious, which can lead to confusion with amyotrophic lateral sclerosis (ALS). Patient 2 had a 6-year duration of the disease, with no fluctuating muscle weakness, a negative neostigmine trial, and appeared to have fasciculations. This patient was easily misdiagnosed as ALS with bulbar-onset symptom. However, her neurological examination and neuroelectrophysiological studies revealed no typical changes in ALS, but a decrement response on low-frequency RNS was revealed in bilateral orbicularis oculi muscles. She then was prescribed with steroid experimental treatment and the QMG score was significantly reduced after the treatment. This response definitively ruled out the suspicion of ALS. Because of the degeneration of ALS motor axon branches and immature collateral regenerative nerves, some ALS can also lead to transmission failure on repetitive stimulation (28). Hence, steroid therapy and the MG-related antibody detection may be the break points to identify the two diseases.

### Other Coexisting ADs and Autoantibodies

Coexisting ADs were detected in 14.6% of AChR-MG patients and in 17.9% of DSN-MG patients, which were consistent with the previous reported total frequency of MG (8–26%) (29, 30). However, the frequency of coexisting AD in MuSK-MG patients was much higher (42.9%). In addition, the frequency of coexisting thyroglobulin (TG) and/or thyroid peroxidase (TPO) antibodies in MuSK-MG patients was 72.7%, which was also higher than the previous reported frequency (15–40%) (31). A Danish study based on the MG population found that MG patients with coexisting AD had a lower rate of disease remission than MG patients without coexisting AD. The study believed that there is a more serious autoimmune reaction in these patients with AD (32). We explored the clinical prognosis between MuSK-MG patients with and without AD, and, specifically, the clinical prognosis between MuSK-MG patients with thyroid antibodies and those without thyroid antibodies. Our results revealed that there was no significant difference in prognosis between the two groups, respectively. In the future, it is still necessary to expand the MuSK-MG sample to verify the relationships between coexisting ADs/thyroid antibodies and prognosis.

### Treatment and Prognosis

Several studies reported that rituximab is an effective immunosuppressant for MuSK-MG patients (19), although this expensive drug is not covered by health insurance in China; thus, no patients received rituximab in our study. Tacrolimus, another effective immunosuppressant drug, has been used for treating AChR-MG (33). In our study, glucocorticoid combined with tacrolimus was prescribed in 13 MuSK-MG patients; however, as the treatment time of some patients in this study was too short, even < year, it may not be appropriate to use the MGFA post-intervention status as the method to evaluate the prognosis. Therefore, we chose the clinical absolute and relative score system for myasthenia gravis as the method to evaluate the therapeutic efficacy, which calculated the percent change of QMG score at each visit from baseline (first visit). Also, no significant difference in the degree of QMG score improvement after treatment was found compared with AChR-MG patients treated with glucocorticoid and an immunosuppressant agent.

This study has several limitations. Comparing clinical outcomes in this case series with other previous reports is difficult because patients were managed differently. In particular, no patients in this study received rituximab. In addition, owing to the small number of MuSK-MG patients and the short follow-up time in a proportion of patients, this study describes the short-term treatment efficacy of MuSK-MG patients in our region. As the results of our study showed that the most severe clinical manifestations in a considerable proportion of MuSK-MG occurred many years after onset, a multi-center study with long-term follow-up is needed in the future. However, the outcomes of tacrolimus treatment in MuSK-MG patients seem to suggest that tacrolimus may not be a bad therapeutic option for those patients, at least in the short term.

In summary, compared with the previous reports, our results provide a distinct understanding of MuSK-MG in terms of age, clinical presentations, and treatment strategy. Patients with MuSK-MG in northeast China have a modestly later onset age and a proportion of patients may have a mild form of the disease with delayed disease progression. We confirmed the existence of a rare ocular MuSK-MG phenotype, a high proportion coexisting with other ADs, and a good response to steroids combined with tacrolimus for our MuSK-MG series.

## Data Availability Statement

The data used to support the findings of this study cannot be shared at this time as the data also forms part of an ongoing study. Requests to access the datasets should be directed to 763595105@qq.com.

## Ethics Statement

The studies involving human participants were reviewed and approved by the ethical committee of Jilin University First Hospital. The patients/participants provided their written informed consent to participate in this study. Written informed consent was obtained from the individual(s) for the publication of any potentially identifiable images or data included in this article.

## Author Contributions

ZZ and YG acquired the clinical data, reviewed the literature, and drafted the article. HD designed the study, supervised the initial drafting, and critically revised the article. JH, ML, and MS analyzed the clinical data and critically revised the article. All authors approved of the final version of the article.

## Conflict of Interest

The authors declare that the research was conducted in the absence of any commercial or financial relationships that could be construed as a potential conflict of interest.
